# Supplementary material for: Mathematical-based morphological classification of skin eruptions corresponding to the pathophysiological state of chronic spontaneous urticaria
Source: Commun Med (Lond). 2023 Dec 4;3:171. doi: 10.1038/s43856-023-00404-8 (PMC10696082; doi:10.1038/s43856-023-00404-8)
Supplement: Supplementary file 2 — Supplementary Information [file 43856_2023_404_MOESM2_ESM.pdf]

Supplementary Information

Mathematical-based morphological classification of skin eruptions corresponding to the pathophysiological state of chronic spontaneous urticaria

Sungrim Seirin-Lee[1,2,3,*], Daiki Matsubara[4], Yuhki Yanase[3,5], Takuma Kunieda[5], Shunsuke Takahagi[3,4,*], and Michihiro Hide[4,6]

[1]Kyoto University Institute for the Advanced Study of Human Biology (ASHBi), Kyoto University, Yoshida-Konoe-cho, Sakyo-ku, Kyoto 606-8501, Japan.

[2]Department of Mathematical Medicine, Graduate School of Medicine, Kyoto University, Yoshida-Konoe-cho, Sakyo-ku, Kyoto 606-8501, Japan.

[3]JST CREST, 4-1-8 Honcho, Kawaguchi, Saitama 332-0012, Japan

[4]Department of Dermatology, Institute of Biomedical & Health Sciences, Hiroshima University, 1-2-3 Kasumi, Minami-ku, Hiroshima 734-8551, Japan

[5]Department of Pharmacotherapy, Graduate School of Biomedical Sciences, Hiroshima University, 1-2-3 Kasumi, Minami-ku, Hiroshima 734-8551, Japan

[6]Department of Dermatology, Hiroshima City Hiroshima Citizens Hospital, Motomachi, Naka-ku, Hiroshima 730-8518, Japan

***Corresponding authors**

Sungrim Seirin-Lee: lee.seirin.2c@kyoto-u.ac.jp

Shunsuke Takahagi: shunstk@hiroshima-u.ac.jp

1. Supplementary Methods

Supplementary method 1: Model description and method for numerical simulations

We recall the CSU model in the main text.

$$\frac{d[H_B]}{dt} = \delta_B + f^B_{activation}([TF])\chi_B(\mathbf{x},t)\left(1 - g^B_{inhibition}([H_B])\right) - \mu_B[H_B] \quad on\ \Omega_B, \quad (1a)$$

$$\frac{d[TF]}{dt} = \delta_T + f^T_{activation}([H_B],[H_M])\left(1 - g^T_{inhibition}([H_B],[H_M])\right) - \mu_T[TF] \quad on\ \Omega_E, \quad (1b)$$

$$\frac{\partial[C]}{\partial t} = D_c\nabla^2 C + f_{leakage}(\mathbf{x},t) - \mu_C[C]\ on\ \Omega_D, \quad (1c)$$

$$\frac{\partial[H_M]}{\partial t} = D_M\nabla^2[H_M] + \delta_M + f^M_{activation}([C])\chi_M(\mathbf{x},t)\left(1 - g^M_{inhibition}([H_M])\right) - \mu_M[H_M] \quad on\ \Omega_D, \qquad (1d)$$

The detailed equations which we used for numerical simulations are given to

$$\frac{d[H_B]}{dt} = \delta_B + \gamma_B[TF]\chi_B(\mathbf{x},t)\left(1 - \frac{\alpha_B[H_B]^2}{\alpha_{B0} + [H_B]^2}\right) - \mu_B[H_B] \quad on\ \Omega_B, \quad (s1a)$$

$$\frac{d[TF]}{dt} = \delta_T + \frac{\gamma_T([H_B]+[H_M])}{\gamma_{T0} + [H_B] + [H_M]}\left(1 - \frac{\alpha_T([H_B]+[H_M])^2}{\alpha_{T0} + ([H_B]+[H_M])^2}\right) - \mu_T[TF] \quad on\ \Omega_E, \quad (s1b)$$

$$\frac{\partial[C]}{\partial t} = D_c\nabla^2 C + \frac{\gamma_C}{1 + \exp(-\beta([TF] - T_{sw}))} - \mu_C[C] \quad on\ \Omega_D, \quad (s1c)$$

$$\frac{\partial[H_M]}{\partial t} = D_M\nabla^2[H_M] + \delta_M + \gamma_M[C]\chi_M(\mathbf{x},t)\left(1 - \frac{\alpha_M[H_M]^2}{\alpha_{M0} + [H_M]^2}\right) - \mu_M[H_M] \quad on\ \Omega_D, \quad (s1d)$$

where $\chi_B(\mathbf{x},t)$ and $\chi_M(\mathbf{x},t)$ are defined by

$$\chi_B(\mathbf{x},t) = \begin{cases} 1 & if\ \int_0^t [H_B](\mathbf{x},\ t)dt \le H_B^{total} \\ 0 & otherwise \end{cases} \quad \text{on } \Omega_B,$$

$$\chi_M(\mathbf{x},t) = \begin{cases} 1 & if\ \int_0^t [H_M](\mathbf{x},\ t)dt \le H_M^{total} \\ 0 & otherwise \end{cases} \quad \text{on } \Omega_D.$$

H_B^{total} and H_M^{total} are the total amounts of histamine contained in basophils and mast cells, respectively. We assume that the total amount of histamine is limited because the time scale of eruption dynamics is from several minutes to hours, which is sufficiently shorter than the time scale of intracellular histamine reproduction (Hattori & Seifert R, 2017; Seirin-Lee, Yanase, et al., 2020). In this model construction, we used TF-mRNA data for the TF activation function on endothelial cell surface, which implicitly assume that the TF expression rate on the cell surface is linearly dependent on the amount of TF-mRNA expression. We also neglect microparticles released from the surface of vascular endothelial cells because it should be immediately diffused in the blood circulation, and, therefore, their contribution should be low, if any, compared with TF on vascular endothelial cells.

We assume that the concentrations of histamine and TF are in stable equilibrium states before external stimulation. External stimuli are represented as spatially random perturbations near equilibrium. The mathematical formulation describing the initial conditions is given by

$$[H_B](\mathbf{x},0) = \frac{\gamma_B}{\mu_B}\big(1 + \epsilon\varphi_1(\mathbf{x})\big) \text{ on } \Omega_B \text{ and } [H_B](\mathbf{x},0) = 0 \text{ on } \Omega_E \setminus \Omega_B,$$

$$[TF](\mathbf{x},0) = \frac{\gamma_T}{\mu_T}\big(1 + \epsilon\varphi_2(\mathbf{x})\big)\ on\ \Omega_E,$$

$$[H_M](\mathbf{x},0) = \frac{\gamma_M}{\mu_M}\big(1 + \epsilon\varphi_3(\mathbf{x})\big)\ and\ [C](\mathbf{x},0) = 0 \text{ on } \Omega_D,$$

where $\varphi_1(\mathbf{x}), \varphi_2(\mathbf{x}),$ and $\varphi_3(\mathbf{x})$ are random valuable functions on [0, 1]and ϵ $(0 < \epsilon < 1)$ is the scale of perturbation which we typically chose 0.01 (1% of equilibrium concentration).

Since we consider our model in the human body, we assume zero-flux boundary conditions. To solve the main equations, we used C programming code written by the Alternating-Direction Implicit (ADI) method (Morton K.W. & Mayers D.F., 1994). The accuracy of the code was confirmed by regenerating examples from published papers and test simulations with smaller grids of time and space.

Supplementary method 2: Data conversion and inhibition functions

To specify the detailed form of the inhibition rate functions to be included in the model $(s1a)$-$(s1d)$, we converted the adenosine concentration data of Fig.2A, B to histamine concentration data. Because the adenosine is released simultaneously with the release of histamine from basophils and hsMCs (Rudich et al., 2012), we assumed that the amount of released adenosine from mast cells or basophils is proportional to the amount of released histamine, namely,

$$[\text{Adenosine concentration }] = \kappa[\text{Histamin concentration}], \qquad (s2)$$

where κ is proportional coefficient. Using this equation, we converted the data of Fig.2A, B with histamine concentrations and estimated the specific forms of $g^B_{inhibition}$ and $g^M_{inhibition}$, as shown in Fig. 2C, by Hill functions such that

$$g^B_{inhibition}([H_B]) = \frac{\alpha_B[H_B]^2}{\alpha_{B0} + [H_B]^2},$$

$$g^M_{inhibition}([H_M]) = \frac{\alpha_M[H_M]^2}{\alpha_{M0} + [H_M]^2}$$

with parameter values, α_B, α_{B0}, α_M, and α_{M0} (See the next section 1.3 for the estimation method in more detail). Similarly, we estimated the inhibition rate function of TF by using data conversion from Fig.2D to the Hill function (Fig.2E),

$$g^T_{inhibition}([H_B],[H_M]) = \frac{\alpha_T([H_B]+[H_M])^2}{\alpha_{T0}+([H_B]+[H_M])^2}.$$

Supplementary method 3: Parameter estimation

Estimation of parameters for the inhibition functions: $g^B_{inhibition}([H_B])$, $g^M_{inhibition}([H_M])$, $g^T_{inhibition}([H_B],[H_M])$

The three inhibitory functions are similar in form. Thus, here we present an estimation method in general form. From the experimental data Figs.2A, 2B, and 2D, we first directly estimated the values of $\hat{\alpha}$ and $\tilde{\alpha}$ by using the following inhibition function of adenosine valuable $[A]$ such that

$$1-\frac{\hat{\alpha}[A]^2}{\tilde{\alpha}+[A]^2}.$$

Then, by instituting the equation $(s2)$ $([A]=\kappa[H])$ into the above equation, we obtained

$$\frac{\hat{\alpha}[A]^2}{\tilde{\alpha}+[A]^2} = \frac{\hat{\alpha}[H]^2}{\frac{\tilde{\alpha}}{\kappa^2}+[H]^2}.$$

Here, we introduce non-dimensional scale of histamine concentration by $[\bar{H}]=[H]/H^*$. That is,

$$\frac{\hat{\alpha}[H]^2}{\frac{\tilde{\alpha}}{\kappa^2}+[H]^2} = \frac{\hat{\alpha}[\bar{H}]^2}{\frac{\tilde{\alpha}}{(\kappa H^*)^2}+[\bar{H}]^2}. \qquad (s3)$$

Next, we tested numerical simulations arbitrarily with changing the value of κH^* and found the proper value at which the eruption pattern reproduced as seen in patients with urticaria. We defined κH^* as a tuning parameter that links *in vitro* experimental data and *in silico* data. In the simulations of this paper, κH^* was approximated to 89.0 with respect to the inhibition functions of $[H_B]$ and $[H_M]$, and $[TF]$. $\hat{\alpha}$ and $\tilde{\alpha}$ were estimated directly from *in vitro* experiments and given to $(\alpha_{B0},\alpha_B)=(50/(\kappa H^*)^2, 0.335)$, $(\alpha_{M0},\alpha_M)=(0.35/(\kappa H^*)^2, 0.865)$, $(\alpha_{T0},\alpha_T)=(71400/(\kappa H^*)^2, 1.0)$ in the mathematical model (s1a)-($s1d$).

Estimation of parameters for activation function: $f^T_{activation}([H_B],[H_M])$

From *in vitro* experimental data of Fig. 2F, we estimated the activation function of TF to the

function of histamine concentration $[H]$.

First, we rescaled the reaction of LPS and Histamine by

$$\sqrt{[H](nM)\times[LPS]\left(\frac{ng}{ml}\right)}\approx\sqrt{[H]^2\bar{\kappa}^2}=[H]\bar{\kappa}, \quad (s4)$$

where we assumed $[LPS]\approx\bar{\kappa}^2[H]$ with a scaling parameter $\bar{\kappa}^2$ of the dimension $[\frac{ng}{ml\times nM}]$. Next, from the data of Fig. 2F, we estimated the function of TF expression with respect to the scaled value of $(s4)$ by

$$[\mathrm{TF-mRNA}]=\frac{b[H]\bar{\kappa}}{a+[H]\bar{\kappa}}=\frac{b[H]}{\frac{a}{\bar{\kappa}}+[H]} \quad (s5)$$

using the least square method. The inferred parameter values are $a/\bar{\kappa}=220$ and $b=1690$. Finally, introducing non-dimensional scales of histamine and TF concentrations by $[\bar{H}]=[H]/H^*$ and $[\overline{TF}]=[TF]/TF^*$with respect to the equation $(s5)$, we have

$$f^T_{activation}([H_B],[H_M])=\frac{\frac{b}{TF^*}[\bar{H}]}{\frac{a}{\bar{\kappa}}H^*+[\bar{H}]}.$$

We chose the values of H^* and TF^* by testing simulations. We arbitrarily changed these values at which the pattern reproduced as seen in patients with urticaria. In the representative simulations, we chose $H^*=10^{-2}$ and TF^*= 402.38. Finally, we obtained $\gamma_T=7.0, \gamma_{T_0}=2.2$.

Supplementary method 4: Modeling descriptions of histamine release in basophils and mast cells: $f^B_{activation}=\gamma_B[TF]$ and $f^M_{activation}=\gamma_M[C]$

The degranulation of hsMCs and basophils is induced by the complement component C5a converted from C5, which is cleaved by coagulation factors triggered by TFs in a concentration-dependent manner (Yanase et al., 2021). Thus, the increasing rate of histamine released from basophil or histamine are written by

$$f^B_{activation} \ or \ f^M_{activation}=\tilde{\gamma}[\text{coagulation factors in intravascular/extravascular}],$$

where $\tilde{\gamma}$ is an increase ratio per unit time. Moreover, the amount of coagulation factors activated by TF in intravascular will be proportional to the expressed TF, namely: $[\text{coagulation factors in intravascular}]=\gamma[TF]$, thus we have $f^B_{activation}=\gamma\tilde{\gamma}[TF]$. Replacing parameter notations, we obtain that

$$f^B_{activation}=\gamma_B[TF] \text{ and } f^M_{activation}=\gamma_M[C],$$

where γ_B is the histamine release rate of basophils by tissue factors and γ_M is the histamine release rate of mast cells induced by the leaked coagulation factors.

Supplementary method 5: Leakage function estimation: $f_{leakage}(\mathbf{x}, t)$

We approximated the increasing rate of leaked coagulation factors in the mathematical model (1c), which is induced by intercellular gap formation driven by a TF-dependent switch-like role:

$$f_{leakage}(\mathbf{x}, t) \propto \frac{d[\text{Area of gap formation}]}{dt}(\mathbf{x}, t) \times \chi_{sw}([TF](\mathbf{x}, t)),$$

where χ_{sw} is a TF-dependent switch-like function.

First, we inferred the TF-dependent switch-like function (χ_{sw}) by calculating the relative CI, $(1 - CI)/(1 - CI_{100\,ng/ml})$, which indicates a relative variation in the magnitude of CI for each time point. We calculated the average of relative CI at [0.2, 0.6] (hour) for each TF concentration and found the switch-like function as shown in Fig. 2I. Based on the result, we propose that the variation of gap formation can be described by a step function, and we defined χ_{sw} as follows (Fig.2J).

$$\chi_{sw}([TF]) = \frac{1}{1 + \exp[-\beta([TF] - T_{sw})]},$$

where T_{sw} is a threshold value of TF at which a cell gap formation is formed, and β is a positive constant expressing the stiffness of switching.

Next, to infer the function of $d[\text{Area of gap formation}]/\text{dt}$, we used the CI data with respect to TF and histamine concentrations (Fig.S1A and C) because the gap area is proportional to the level of CI. Then we obtain the following result, as shown in Fig. S1B and D:

$$\frac{d}{dt}[\text{Area of gap formation}](\cdot, t) \propto \frac{\Delta[CI](t)}{\Delta t} \approx 0.$$

Hence, we obtain $[\text{Area of gap formation}](\cdot, t) \approx \gamma_{TF} + \gamma_{Histamine} = \gamma_C$ (constant). This estimation indicates that the gap formation may quickly approach the size limit, independent of TF and histamine concentrations if the TF state is switched to ON. Thus, the TF switch-like dynamics may play a key role in onset/non-onset of CSU.

Supplementary method 6: Statical analysis for patients' data

Verification of total sample number for classification

We classified the eruption types into six categories: annual type, broken-annual type, geographic type, circular type, dot type, unclassifiable type. Based on the statical analysis of (Thompson, 1987), the minimal number of samples required for the error with the true classification result to be within 8 % with the significance level (α) of 5 % is given to 94 samples. The number of total

samples which we used in this study is 105 patient samples.

Verification of classifiable ratio 87.6 %

To confirm that the classifiable ratio 87.6% is not rare event with respect to numerous sample data, we used P-value test. Let us assume that the null hypothesis (H_0); The classifiability of the population by EGe Criteria is no higher than 79%, and the alternative hypothesis (H_1); The classifiability of the population by EGe Criteria exceeds 79%. We then obtain $P_{value} = \sum_{k=k_0}^{n} nC_k\, p_0^k (1-p_0)^{n-k} < 0.016$ where $k_0 = 92, n = 105,$ and $p_0 = 0.79$. Thus, we adopt H_1 depending on the significance level (α=2.5 %) of the one-tailed test.

Supplementary method 7: Sensitivity analysis

For the sensitivity analysis, we used the variance-based sensitivity analysis by using Extended Fourier Amplitude Sensitivity Test (eFAST) Method (See Appendix A.3 of (Seirin-Lee, Gaffney, et al., 2020) for the details of the method). For the calculations of the first order sensitivity index and the total-effect index, we used the frequencies {19, 23, 59, 77, 91, 107, 113, 121, 125, 133, 149, 157, 161} and {1, 1, 3, 5, 7, 9, 11, 13, 15, 17, 19, 21, 168}, respectively. Thus, the total sample size, $N_s = (2M\omega_{max} + 1)N_r$, is given to 1289 and 1345, respectively, with $M = 4, N_r = 2$.

We calculated two sensitivity measures, the first order index and total-effect index. The first order index indicates the influence of parameter p_i on the variance of each feature measure (the model output), independent of interactions with the other parameters. The total-effect index indicates the effect of parameter/network p_i when interactions with the other parameters/networks are included. These two measures give a full quantification of the importance of parameter/network p_i and whether the extent whether this is a direct influence or through interactions with other parameters/networks or both. We show the representative result of the first order index in Fig.6A-E. The similar results are shown in the total-effect index (Fig. S2).

Based on each feature of wheal patterns in Table 2, we defined the sensitivity function (S_f) for each type of eruption patterns as the following.

[Boundary: KF1]

We defined the sensitivity function which give how much the boundary property is maintained,

as the following.

When

$$Bd \equiv \left| \int_0^L \int_0^L |S_w(x,y,t^*) - S_w(x,y,t_0)| dxdy - \int_0^L \int_0^L |S_w(x,y,t^*)| dxdy - \int_0^L \int_0^L |S_w(x,y,t_0)| dxdy \right| < \mathcal{E},$$

$$S_f = \left| \int_0^L S_w\left(x, \frac{L}{2}, t^*\right) - S_w\left(x, \frac{L}{2}, t_0\right) dx \right|.$$

[Boundary-disconnected: KF2]

We defined the sensitivity function which give how much the disconnected property of boundary wheal is promoted, as the following.

When $Bd < \varepsilon$,

$$S_f = \frac{1}{2}\left\{ \left| \int_0^L \int_0^{L/2} S_w(x,y,t^*) dxdy - \int_0^L \int_{L/2}^L S_w(x,y,t^*) dxdy \right| + \left| \int_0^L \int_0^{L/2} S_w(x,y,t^*) dydx - \int_0^L \int_{L/2}^L S_w(x,y,t^*) dydx \right| \right\}$$

[Area: KF3]

We defined the sensitivity function which give how much the area property is maintained, as the following.

When

$$Ar \equiv \int_0^L \int_0^L |S_w(x,y,t^*) - S_w(x,y,t_0)| - \int_0^L \int_0^L |S_w(x,y,t^*)| < 0,$$

$$S_f = \left| \int_{\frac{L}{2}-\delta_\varepsilon}^{\frac{L}{2}+\delta_\varepsilon} \int_{\frac{L}{2}-\delta_\varepsilon}^{\frac{L}{2}+\delta_\varepsilon} S_w(x,y,t^*) dxdy - \int_{\frac{L}{2}-\delta_\varepsilon}^{\frac{L}{2}+\delta_\varepsilon} \int_{\frac{L}{2}-\delta_\varepsilon}^{\frac{L}{2}+\delta_\varepsilon} S_w(x,y,t_0) dxdy \right| \frac{1}{t^* - t_0}$$

where δ_ε is a small radius.

[Area fusion: KF4]

To determine the extent to which area-type wheals fuse, we defined a sensitivity function that gives the degree of area weal expansion as follows.

When $Ar < 0$,

$$S_f = \frac{1}{t^* - t_0} \sum_{t=t_0}^{t^*} \frac{1}{\Delta t} \left| \int_0^L \int_0^L S_w(x,y,t+\Delta t)\, dxdy - \int_0^L \int_0^L S_w(x,y,t) dxdy \right|$$

[Punctate: KF5]

We defined the sensitivity function which give how much the small and fixed size wheal is maintained, as the following.

When

$$Dot \equiv \int_0^L \int_0^L S_w(x,y,t^*)\, dxdy < \varepsilon_0,$$

$$S_f = \sum_{t=0}^{t^*} \int_0^L \int_0^L S_w(x,y,t)\, dxdy.$$

To calculate the first order sensitivity index and the total-effect index, we chose initial conditions by which a wheal pattern emerges in the center area of simulation space. The example is shown in Fig. S3G. The results of total index are shown in Fig. S3A-F. In the case of the total-effect index of broken-boundary, the index value of dummy parameter was higher than all the other model parameters, so that we could not obtain any valid information. Nonetheless, we found that the effect of the positive networks for histamine release from basophils (γ_B, δ_B) are negligible, indicating that basophils may not play an important role in the variation of wheal shapes and be involved in the triggering phase of CSU development.

Supplementary method 8: Simulation space and numerical method

We simulated a two-dimensional (2D) model of the dermal region (Ω_D) and vascular endothelial tissue (Ω_E), whereas $\Omega_B (\subset \Omega_E)$ is a random region of vascular endothelial tissue where histamine released from basophils affected endothelial cells. In model simulations, we set

$$\Omega_D = \Omega_E = [0, L] \times [0, L] \text{ and}$$

$$\Omega_B = \left\{ \mathbf{x} \middle| \mathbf{x} = \mathrm{RAND}([0, L] \times [0, L]),\ [H_B](\mathbf{x}, 0) > \frac{\gamma_B}{\mu_B}\big(1 + \epsilon \theta_b \varphi_1(\mathbf{x})\big) \right\},$$

where RAND is the random value function of the uniform distribution and $\theta_b \in [0,1]$ is the threshold level at which initial stimulation by endothelial cell contact of basophils affect the vascular endothelial tissue. The numerical simulation code was written using C language based

on the alternating-direction implicit (ADI) method.

References

1. Hattori, Y., & Seifert R. Histamine and Histamine Receptors in Health and Disease (Part IV: Church MK, Allergy, histamine and antihistamines). 2017; *Springer, Cham*.
2. Morton K.W., & Mayers D.F. *Numerical Solution of Partial Differential Equations.* 1994; *Cambridge University Press*.
3. Rudich N, Ravid K, Sagi-Eisenberg R. Mast cell adenosine receptors function: A focus on the A3 adenosine receptor and inflammation. *Front Immunol* 2012;3:134. https://doi.org/10.3389/fimmu.2012.00134
4. Seirin-Lee, S., Gaffney, E. A., & Dawes, A. T. CDC-42 Interactions with Par Proteins Are Critical for Proper Patterning in Polarization. *Cells*, 2020;*9*(9). https://doi.org/10.3390/cells9092036
5. Seirin-Lee, S., Yanase, Y., Takahagi, S., & Hide, M. (2020). A single reaction-diffusion equation for the multifarious eruptions of urticaria. *PLoS Computational Biology*, *16*(1), e1007590. https://doi.org/10.1371/journal.pcbi.1007590
6. Thompson, S. K. *Sample Size for Estimating Multinomial Proportions, 1987;* 41; Issue 1.
7. Yanase Y, Matsuo Y, Takahagi S, Kawaguchi T, Uchida K, Ishii K, et al. Coagulation factors induce human skin mast cell and basophil degranulation via activation of complement 5 and the C5a receptor. *J Allergy Clin Immunol* 2021;147:1101-4. https://doi.org/10.1016/j.jaci.2020.08.018

1 Supplementary figures and tables

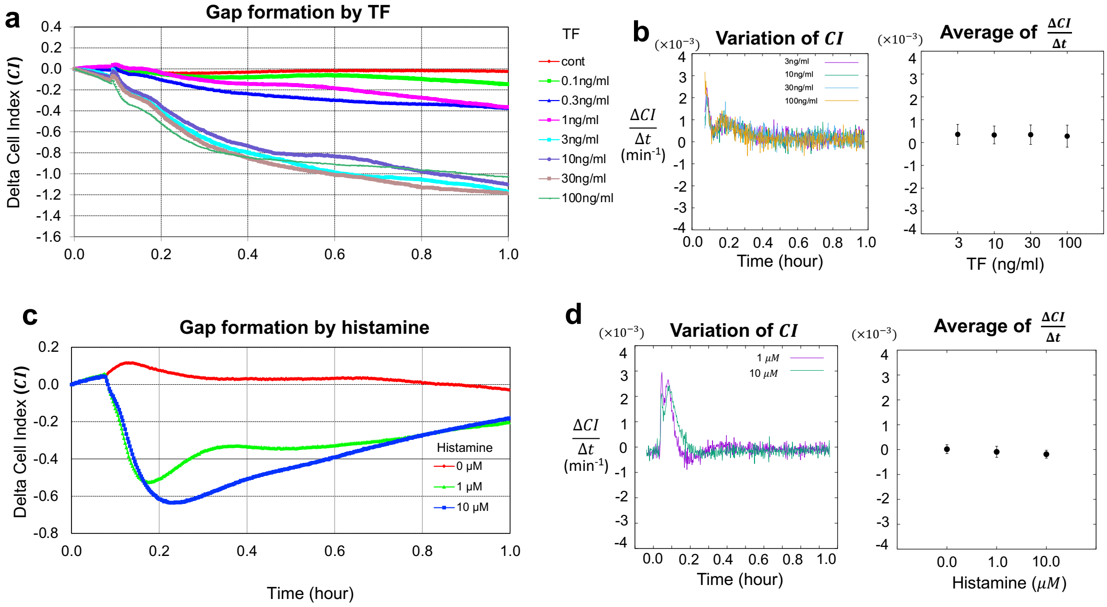

Fig.S1: Gap formation effect by Tissue factor (TF) and histamine. (**a**) Temporal variation in cell index (CI) at different TF concentrations. (**b**) Quantitative analysis of variation in CI at different TF concentrations. (**c**) Temporal variation in CI with varying histamine concentrations. Endothelial cells were stimulated with histamine at indicated concentrations in DMEM/F12 supplemented with 10% fetal calf serum (FCS). (**d**) Quantitative analysis of CI variation for histamine concentrations.

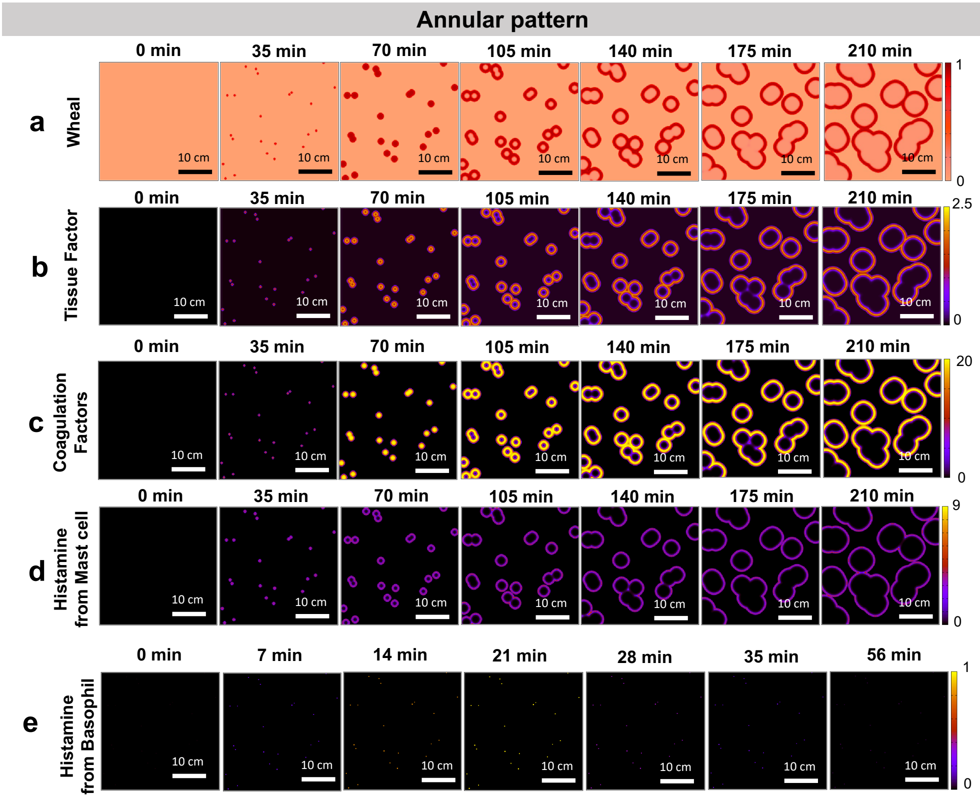

Fig.S2: Dynamics of annular wheal patterns on skin (**a**), tissue factor expressed on vascular endothelial cells ($[TF]$) (**b**), coagulation factors leaked from blood vessel ($[C]$) (**c**), histamine released from mast cells ($[H_M]$) (**d**), and histamine released from basophils ($[H_B]$) (**e**) for each type of patterns.

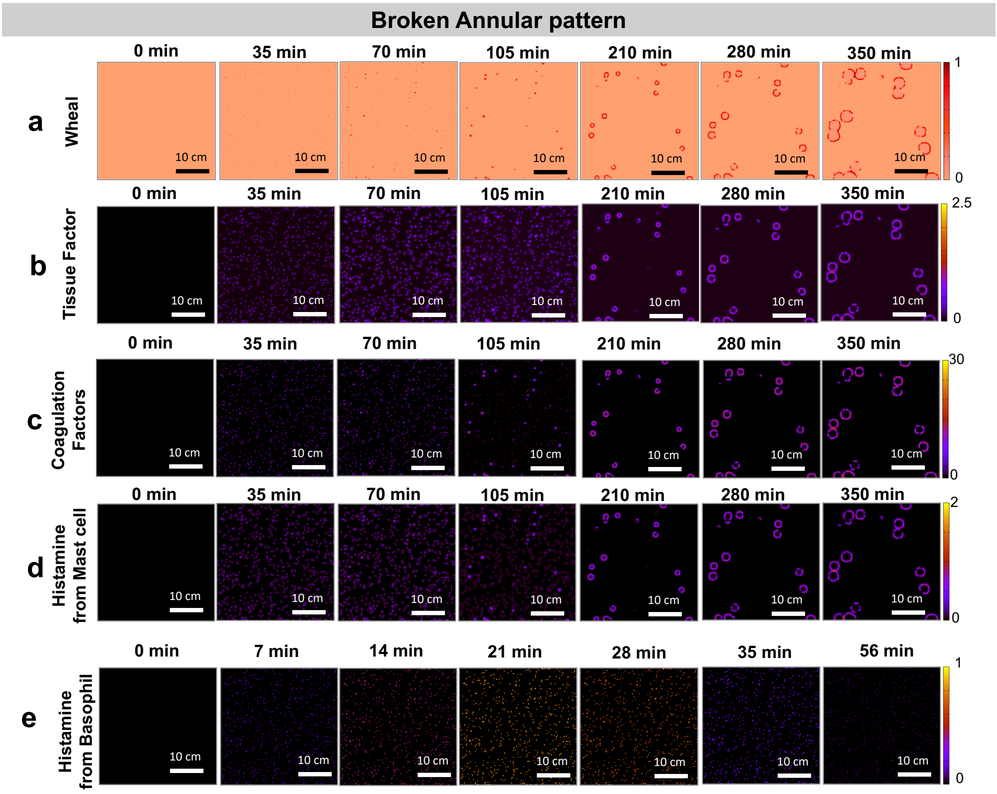

Fig.S3: Dynamics of broken-annular wheal patterns on skin (**a**), tissue factor expressed on vascular endothelial cells ($[TF]$) (**b**), coagulation factors leaked from blood vessel ($[C]$) (**c**), histamine released from mast cells ($[H_M]$) (**d**), and histamine released from basophils ($[H_B]$) (**e**) for each type of patterns.

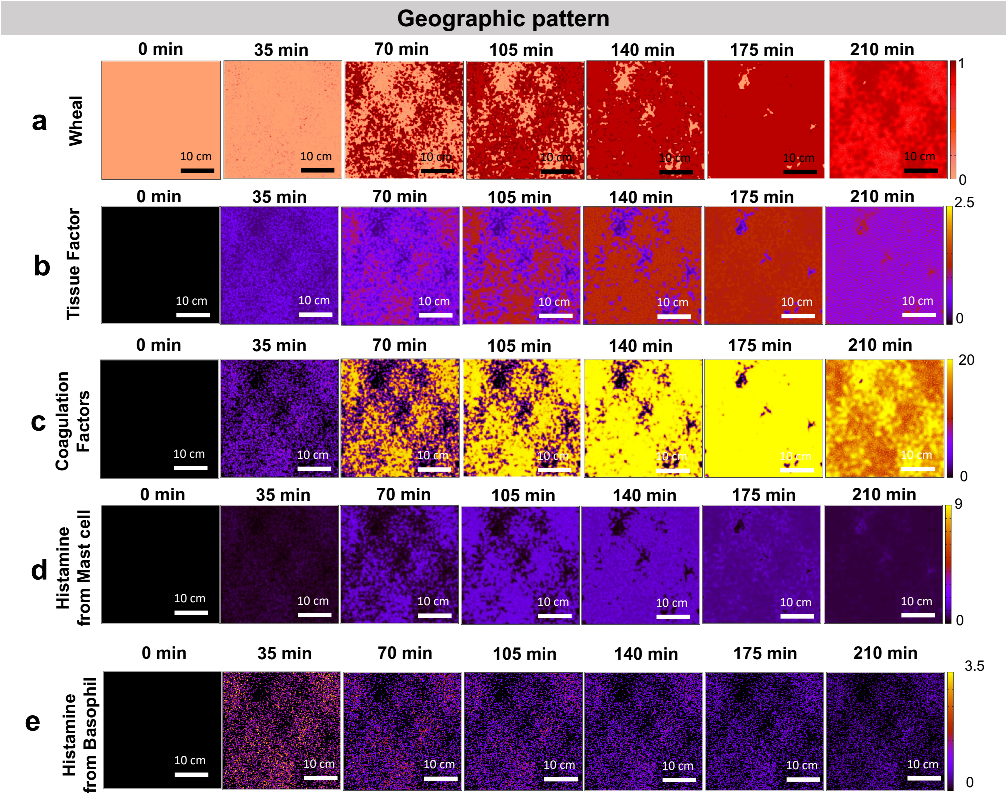

Fig.S4: Dynamics of geographic wheal patterns on skin (**a**), tissue factor expressed on vascular endothelial cells ($[TF]$) (**b**), coagulation factors leaked from blood vessel ($[C]$) (**c**), histamine released from mast cells ($[H_M]$) (**d**), and histamine released from basophils ($[H_B]$) (**e**) for each type of patterns.

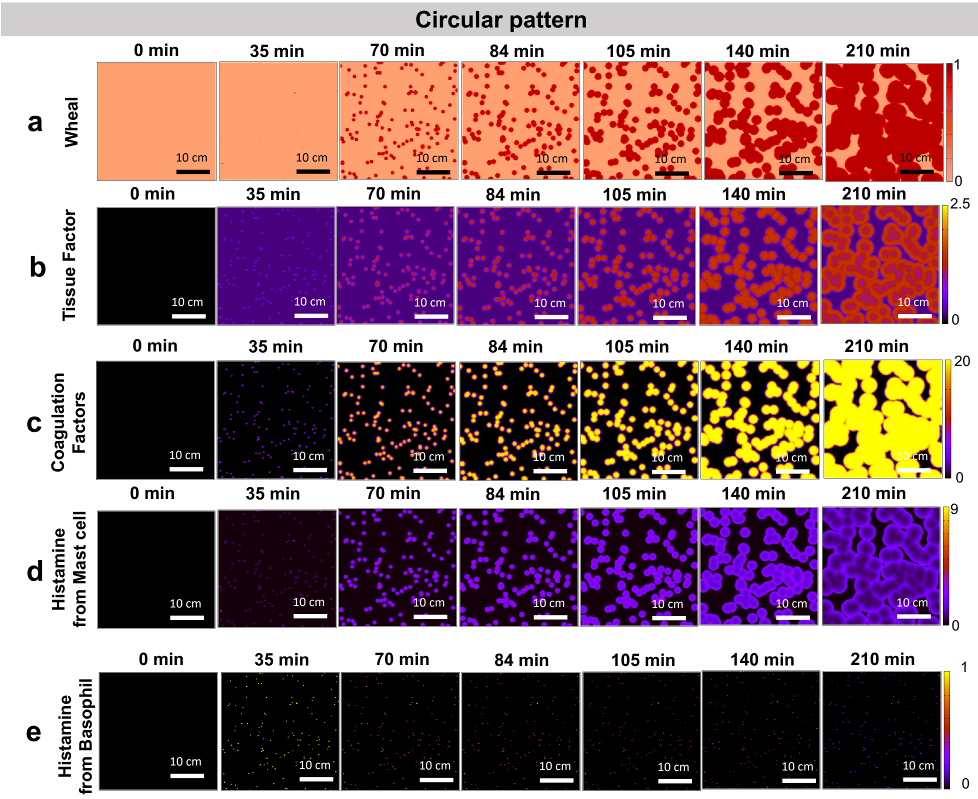

Fig.S5: Dynamics of circular wheal patterns on skin (**a**), tissue factor expressed on vascular endothelial cells ($[TF]$) (**b**), coagulation factors leaked from blood vessel ($[C]$) (**c**), histamine released from mast cells ($[H_M]$) (**d**), and histamine released from basophils ($[H_B]$) (**e**) for each type of patterns.

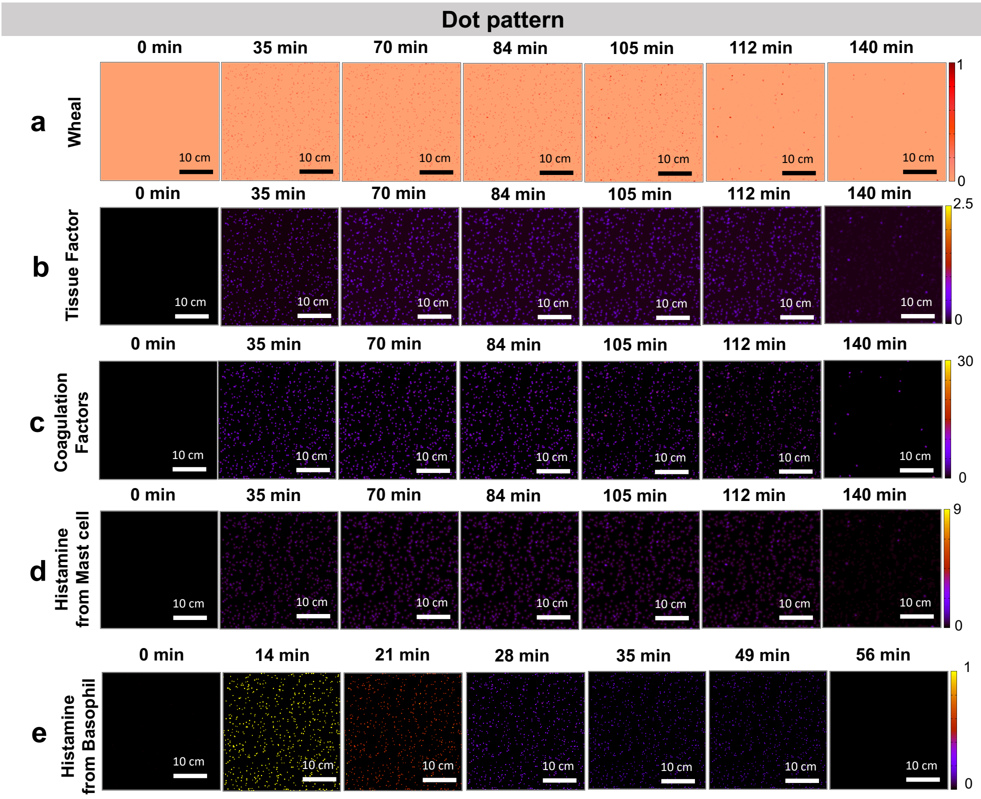

Fig.S6: Dynamics of dot wheal patterns on skin (**a**), tissue factor expressed on vascular endothelial cells ($[TF]$) (**b**), coagulation factors leaked from blood vessel ($[C]$) (**c**), histamine released from mast cells ($[H_M]$) (**d**), and histamine released from basophils ($[H_B]$) (**e**) for each type of patterns.

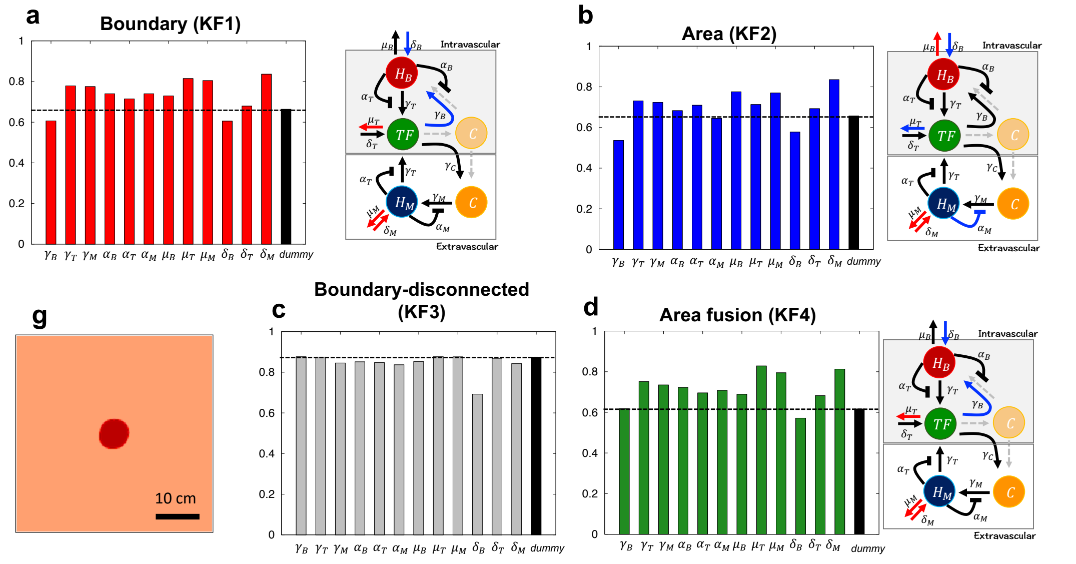

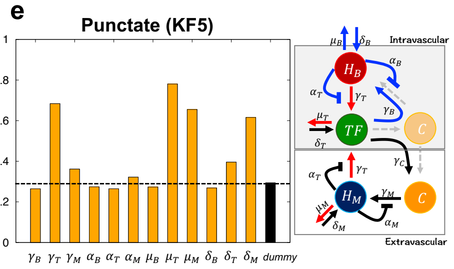

f Legend: ✓ Effect present; ✓ (highlighted) Top 3 High effect; grey cell: Effect negligible

	Basophils				Tissue Factor				Mast cell			
	γ_B	α_B	μ_B	δ_B	γ_T	α_T	μ_T	δ_T	γ_M	α_M	μ_M	δ_M
Boundary (**KF1**)		✓	✓		✓	✓	✓ (Top 3)	✓	✓	✓	✓ (Top 3)	✓ (Top 3)
Boundary-disconnected (**KF2**)												
Area (**KF3**)		✓	✓ (Top 3)		✓	✓	✓	✓	✓	✓	✓ (Top 3)	✓ (Top 3)
Area fusion (**KF4**)		✓	✓		✓	✓	✓ (Top 3)	✓	✓	✓	✓ (Top 3)	✓ (Top 3)
Punctate (**KF5**)					✓ (Top 3)		✓ (Top 3)	✓	✓	✓	✓ (Top 3)	✓

Fig.S7: Result for the total-effect index (a-f) and the example simulation used for the sensitivity analysis (g). A representative parameter value set in Table S1 was used for analysis. Red/blue arrows indicate high/negligible effects. Since none of the total-effect indices was markedly different from the dummy parameter case for KF1-KF5, the arrows for the top three total-effect indices are indicated in red.

Table S1. The details of parameter values for each eruption pattern used in simulations and the representative parameter value set for sensitivity analysis. Parameter values estimated from the experimental data in Fig. S2 is noted by asterisk (*). Diffusion coefficient of histamine (D_M) was estimated by clinical experiment data in our previous study (Seirin-Lee, Yanase, et al., 2020). Dimension values are shown in parentheses. [ ] represents the parameter range which used for sensitivity analysis.

Parameters	Representative Value Set	Annular	Broken annular
Spatial length ($L \times L$)	1×1 ($35.5 \times 35.5 \mathrm{cm}^2$)	1×1	1×1
Time scale (t)	1.0 (420 sec)	1.0	1.0
Diffusion rates of histamine (D_M) and coagulation factors (D_C)	4.7×10^{-6} (1.412×10^{-5} $\mathrm{cm}^2/\mathrm{sec}$)*	4.7×10^{-6} (1.412×10^{-5} $\mathrm{cm}^2/\mathrm{sec}$)*	4.7×10^{-6} (1.412×10^{-5} $\mathrm{cm}^2/\mathrm{sec}$)*
Basal secretion rates of histamine from basophil (δ_B)	[0, 1.0] ([0.0, 0.0024] sec^{-1})	0.1 (0.00024 sec^{-1})	0.1 (0.00024 sec^{-1})
Histamine release rate of basophil (γ_B)	[0, 5.0] ([0.0, 0.012] sec^{-1})	5.0 (0.012 sec^{-1})	5.0 (0.012 sec^{-1})
Parameter affecting the gradient of inhibition rate of histamine from basophil (α_{B0})	0.00625 *	0.00625 *	0.00625 *
Maximal inhibition rate of histamine from basophil (α_B)	[0, 1.0]	0.335 *	0.335 *
Basal decay rate of histamine from basophil (μ_B)	[0, 1.0] ([0.0, 0.0024] sec^{-1})	1.0 (0.0024 sec^{-1})	1.0 (0.0024 sec^{-1})
Basal secretion rates of tissue factor (δ_T)	[0, 1.0] ([0.0, 0.0024] sec^{-1})	0.01 (0.000024 sec^{-1})	0.01 (0.000024 sec^{-1})
Maximal increase rate of tissue factor (γ_T)	4.2 * [0, 5.0] ([0.0, 0.012] sec^{-1})	7.0 (0.017 sec^{-1})	4.2 * (0.01 sec^{-1})
Parameter determining the curve of increasing tissue factor (γ_{T0})	2.2 *	2.2 *	2.2 *
Basal decay rate of tissue factor (μ_T)	[0, 1.0] ([0.0, 0.0024] sec^{-1})	1.0 (0.0024 sec^{-1})	1.0 (0.0024 sec^{-1})
Parameter affecting the gradient of inhibition rate of tissue factor	8.925 *	8.925 *	8.925 *

(α_{T0})			
Maximal inhibition rate of histamine from basophil (α_T)	1.0 * [0, 1.0]	1.0 *	1.0 *
Leakage rate of coagulation factors from blood vessel (γ_C)	20.0 (0.0476 sec^{-1})	20.0 (0.0476 sec^{-1})	20.0 (0.0476 sec^{-1})
Parameter determining the stiffness of switching (β)	20.0	20.0	20.0
Switch value of tissue factor (T_{sw})	0.67	0.67	0.67
Basal decay rate of coagulation factors (μ_C)	[0, 1.0] ([0.0, 0.0024] sec^{-1})	1.0 (0.0024 sec^{-1})	1.0 (0.0024 sec^{-1})
Basal secretion rates of histamine from mast cell (δ_M)	[0, 1.0] ([0.0, 0.0024] sec^{-1})	0.01 (0.000024 sec^{-1})	0.01 (0.000024 sec^{-1})
Histamine release rate of mast cell (γ_M)	[0, 5.0] ([0.0, 0.012] sec^{-1})	5.0 (0.012 sec^{-1})	0.8 (0.002 sec^{-1})
Parameter affecting the gradient of inhibition rate of histamine from mast cell (α_{M0})	4.37×10^{-5} *	4.37×10^{-5} *	4.37×10^{-5} *
Maximal inhibition rate of histamine from basophil (α_M)	0.865 * [0, 1.0]	0.865 *	0.865 *
Basal decay rate of histamine from mast cell (μ_M)	[0, 1.0] ([0.0, 0.0024] sec^{-1})	1.0 (0.0024 sec^{-1})	1.0 (0.0024 sec^{-1})
Total amount of histamine of basophil (H_B^{total})	600	600	600
Total amount of histamine of mast cell (H_M^{total})	600	600	600
Parameter determining the stiffness of wheal state function (β_w)	1.0	1.0	1.0

**Parameters**	**Geographic**	**Circular**	**Dot**
Spatial length ($L \times L$)	1×1 ($35.5 \times 35.5\text{cm}^2$)	1×1 ($35.5 \times 35.5\text{cm}^2$)	1×1 ($35.5 \times 35.5\text{cm}^2$)
Time scale (t)	1.0 (420 sec)	1.0 (420 sec)	1.0 (420 sec)
Diffusion rates of histamine (D_M) and coagulation factors (D_C)	4.7×10^{-6} (1.412×10^{-5} cm^2/sec) *	4.7×10^{-6} (1.412×10^{-5} cm^2/sec) *	4.7×10^{-6} (1.412×10^{-5} cm^2/sec) *
Basal secretion rates of histamine from basophil	0.1	0.1	0.5

(δ_B)	(0.00024 sec^{-1})	(0.00024 sec^{-1})	(0.0012 sec^{-1})
Histamine release rate of basophil (γ_B)	5.0<br>(0.012 sec^{-1})	5.0<br>(0.012 sec^{-1})	20.0<br>(0.048 sec^{-1})
Parameter affecting the gradient of inhibition rate of histamine from basophil (α_{B0})	0.00625 *	0.00625 *	0.00625 *
Maximal inhibition rate of histamine from basophil (α_B)	0.335 *	0.335 *	0.335 *
Basal decay rate of histamine from basophil (μ_B)	1.0<br>(0.0024 sec^{-1})	1.0<br>(0.0024 sec^{-1})	1.0<br>(0.0024 sec^{-1})
Basal secretion rates of tissue factor (δ_T)	0.01<br>(0.000024 sec^{-1})	0.01<br>(0.000024 sec^{-1})	0.01<br>(0.000024 sec^{-1})
Maximal increase rate of tissue factor (γ_T)	4.2 *<br>(0.01 sec^{-1})	4.2 *<br>(0.01 sec^{-1})	4.2 *<br>(0.01 sec^{-1})
Parameter determining the curve of increasing tissue factor (γ_{T0})	2.2 *	2.2 *	2.2 *
Basal decay rate of tissue factor (μ_T)	1.0<br>(0.0024 sec^{-1})	1.0<br>(0.0024 sec^{-1})	1.0<br>(0.0024 sec^{-1})
Parameter affecting the gradient of inhibition rate of tissue factor (α_{T0})	8.925 *	8.925 *	8.925 *
Maximal inhibition rate of histamine from basophil (α_T)	1.0 *	1.0 *	1.0 *
Leakage rate of coagulation factors from blood vessel (γ_C)	20.0<br>(0.0476 sec^{-1})	20.0<br>(0.0476 sec^{-1})	20.0<br>(0.0476 sec^{-1})
Parameter determining the stiffness of switching (β)	20.0	20.0	20.0
Switch value of tissue factor (T_{sw})	0.67	0.67	0.67
Basal decay rate of coagulation factors (μ_C)	1.0	1.0	1.0
Basal secretion rates of histamine from mast cell (δ_M)	0.01<br>(0.000024 sec^{-1})	0.01<br>(0.000024 sec^{-1})	0.01<br>(0.000024 sec^{-1})
Histamine release rate of mast cell (γ_M)	0.2<br>(0.00048 sec^{-1})	1.0<br>(0.0024 sec^{-1})	1.0<br>(0.0024 sec^{-1})
Parameter affecting the gradient of inhibition rate of histamine from mast cell (α_{M0})	4.37×10^{-5} *	4.37×10^{-5} *	4.37×10^{-5} *
Maximal inhibition rate			

of histamine from basophil (α_M)	0.865 *	0.865 *	0.865 *
Basal decay rate of histamine from mast cell (μ_M)	0.1 (0.00024 sec^{-1})	0.1 (0.00024 sec^{-1})	0.1 (0.00024 sec^{-1})
Total amount of histamine of basophil (H_B^{total})	600	600	600
Total amount of histamine of mast cell (H_M^{total})	600	600	600
Parameter determining the stiffness of wheal state function (β_w)	1.0	1.0	1.0

Table S2. Mathematical classification of eruption patterns corresponding to the EGe criteria. We define a set of a single wheal region by $B_s(t)$ and the sets of total wheal regions by $B_{total}(t) = \bigcup_s B_s(t) \equiv \{\mathbf{x} \in \Omega | S_w(\mathbf{x}, t) = 1\}$ for $t_e < t < t_d$. $s \in \mathbb{Z}^+$is the index of wheals. t_e and t_d are the time points at which eruption patten is emerged and disappeared, respectively. $t_1, t_2 \in [t_e, t_d]$.

<table>
<tr><th>Class</th><th>Math Feature</th><th>Type</th><th>Math Feature</th></tr>
<tr><td rowspan="2">Boundary pattern</td><td rowspan="2">$\exists t_1, t_2$ $such\ that$ $B_s(t_1) \not\subset B_s(t_2)$ for $t_1 < t_2$</td><td>Annular pattern</td><td>$\Omega \setminus B_{total}(t)$ is a disconnected set;
✓ Ring-shape or connected multiple arcs.
✓ Multiple annular patterns are fused and make bigger one.</td></tr>
<tr><td>Broken annular pattern</td><td>$\Omega \setminus B_{total}(t)$ is a connected set;
✓ Ring-shape broken in part or disconnected multiple arc shape for $B_s(t)$.
✓ Disconnected state of $B_s(t)$ is retained even after fusions.</td></tr>
<tr><td rowspan="3">Area pattern</td><td rowspan="3">$B_s(t_1) \subset B_s(t_2)$ for $\forall t_1 < t_2$</td><td>Geographic pattern</td><td>✓ The expansion speed of $B_s(t)$ is relatively rapid and $B_s(t)$ has a complicated boundary shape.</td></tr>
<tr><td>Circular pattern</td><td>✓ $B_s(t)$ is close to the circular shape. Each $B_s(t)$ are relatively independent and have no rapid coalescence during the period $t_e < t < t_d$.</td></tr>
<tr><td>Dot pattern</td><td>$B_s(t) \cap B_p(t) = \emptyset\ for\ \forall s \neq p$, and $|B_s(t)| \ll 1$;
✓ Dot state.
✓ No coalescence of wheals is seen.</td></tr>
</table>
