# Supplementary material for: Mathematical-based morphological classification of skin eruptions corresponding to the pathophysiological state of chronic spontaneous urticaria
Source: Commun Med (Lond). 2023 Dec 4;3:171. doi: 10.1038/s43856-023-00404-8 (PMC10696082; doi:10.1038/s43856-023-00404-8)
Supplement: Supplementary file 3 — Description of Additional Supplementary Files [file 43856_2023_404_MOESM3_ESM.pdf]

Description of Additional Supplementary Files

File Name: Supplementary Data 1
Description: Supplementary Data 1 contains source data for the main figures (Fig.2 and Fig.4)
